# Dual-interventional therapy for multiple splenic artery aneurysms in a patient with portal hypertension: A case report

**DOI:** 10.1097/MD.0000000000015205

**Published:** 2019-05-13

**Authors:** Hongtao Niu, Sha Junfeng, An Jianli, Zou Zibo, Dong Yanchao

**Affiliations:** Department of Interventional Radiology, The First Hospital of Qinhuangdao, Qinhuangdao, Hebei, P.R. China.

**Keywords:** embolization, portal hypertension, splenic artery aneurysm (SAA), transjugular intrahepatic portosystemic shunt (TIPS) ;

## Abstract

**Rationale:**

Portal hypertension (PHT) is either a significant risk factor of development of splenic artery aneurysm (SAA), or predisposing factor of rupture.

**Patient concerns:**

A 57-year-old patient was admitted to our hospital because of multiple SAAs with PHT, suffered from episodes of haematemesis.

**Diagnosis:**

Emergency ultrasound of the abdomen showed remarkable cirrhosis and splenomegaly. Two days later, CT angiography reveal two SAA located in the splenic artery, as well as splenomegaly and features of PHT.

**Interventions:**

Transjugular intrahepatic portosystemic shunt (TIPS) was performed to decrease portal venous pressure and control esophagogastric variceal hemorrhage. Coil embolization of the main splenic artery was performed to complete thrombosis of the two SAAs and relieve critical hypersplenism.

**Outcomes:**

After 3 months, follow-up enhanced CT confirmed complete thrombosis of the main splenic artery and the two aneurysm sac, and partial splenic infarction (approximately 50%).

**Lessens:**

TIPS can control easophagogastric variceal hemorrhage and decrease portal venous pressure, coil embolization of the main splenic artery can promote permanent thrombosis of aneurysm sac and relieve hypersplenism.

## 1 Introduction

Splenic artery aneurysms (SAAs) are the commonest of all visceral aneurysms accounting for an incidence of 0.1% in the general population. Portal hypertension (PHT) is a significant risk factor for the development of an SAAs.^[1]^ Furthermore, the risk of rupture in SAAs is reported to be higher in patients with PHT than in patients without such a history (56% vs 17%, respectively).^[2]^

The most frequent management options for SAAs are medical treatment, open or laparoscopic surgery, and endovascular treatment. Endovascular treatment of SAAs is increasingly being used over surgery in the elective setting.^[3]^ After unsuccessful endoscopic procedure for acute gastroesophageal variceal bleeding resulted from PHT, transjugular intrahepatic portosystemic shunt (TIPS) placement has become an established procedure in China, due to lack of liver donor or poor economic condition. We report a patient with multiple SAAs and PHT who presented with gastroesophageal variceal bleeding and was treated with combined TIPS and coil embolization of the splenic artery.

## 2 Case presentation

**Figure 1:**
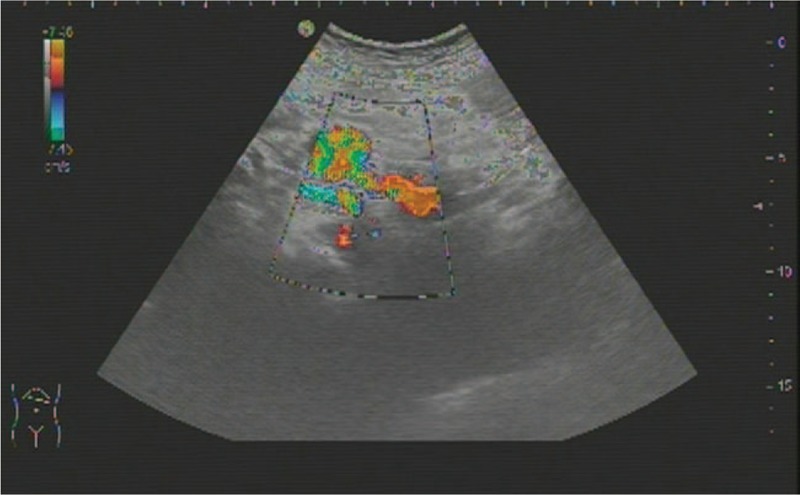
Emergency Abdominal ultrasound (US) with Doppler studies reveals a vascular, arterial mass (2.7 × 2.0 cm) in the proximal of splenic artery, blue and red Doppler signals within the mass demonstrate turbulent blood flow. But missed another smaller SAA near the splenic hilum.

**Figure 2:**
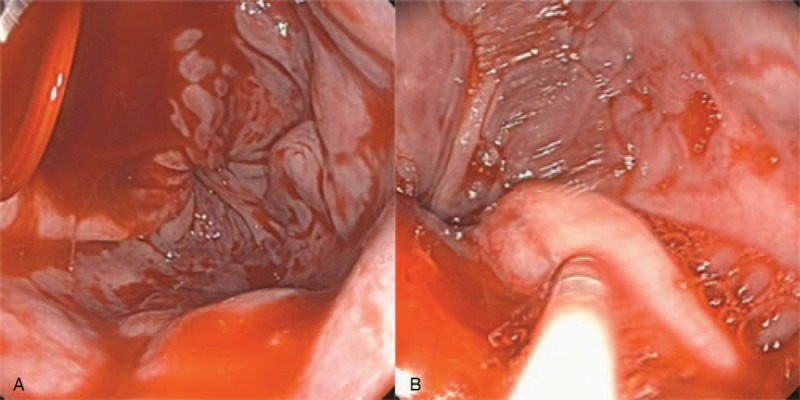
Urgent oesophagogastroduodenoscopy revealed large oesophageal varices in lower esophageal and active bleeding, lauromacrogol, and human tissue glue were injected into varices to control hemorrhage immediately.

**Figure 3:**
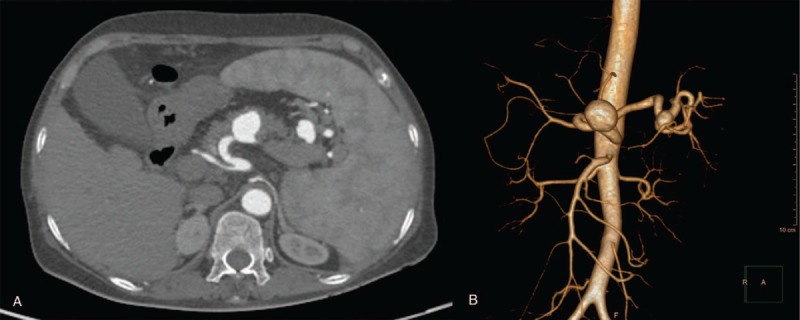
Axial 1.5-mm section and 3D reconstruction from a CT angiogram depicting one larger wide-necked aneurysm in the proximal splenic artery and another smaller SAA near the splenic hilum.

A 57-yearold woman with known PHT due to chronic hepatitis B was admitted to the emergency room with acute onset of a large variceal bleed. On examination the patient was pale, hypotensive, sinus tachycardic, and mild tenderness in the left upper quadrant. Blood examination showed low total leukocyte (1.6 × 10/L), hemoglobin (82 g/L) and platelet counts (63 × 10/L). Liver function grade was classified into Child B. Emergency ultrasound (US) of the abdomen showed remarkable cirrhosis and splenomegaly, but no evidence of ascites. An echolucent lesion (2.7 × 2.0 cm) was identified in the proximal of splenic artery. A color Doppler study suspected this to be an aneurysm (Fig. 1). After initial resuscitation and stabilization of the patient's status, urgent oesophagogastroduodenoscopy was carried out, which showed significant bleeding oesophageal varices that were treated with sclerotherapy (Fig. 2). The stomach was found full of blood and the gastric mucosa was red irritated and edematous; which was consistent with portal gastritis. Two days later, computer tomography (CT) angiography was performed to reveal one larger wide-necked SAA (2.7 × 2.3 cm) located in the proximal of splenic artery, another smaller SAA (1.7 × 1.4 cm) near the splenic hilum, as well as splenomegaly and features of PHT (Fig. 3).

**Figure 4:**
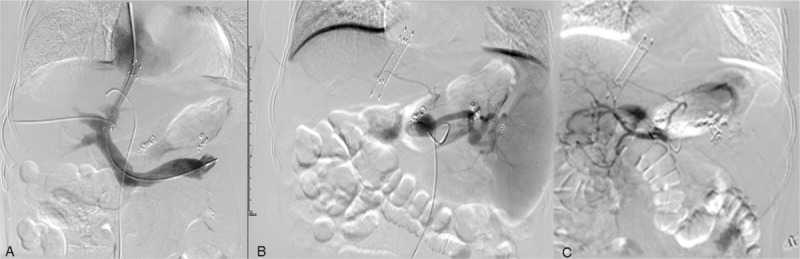
A: Digital subtraction angiography of the splenic vein shows one bare stent and one covered stent were overlapped to createshunt between the middle hepatic vein and left portal vein. Percutaneous transhepatic coronary vein and short gastric veins were occluded to control hemorrhage. B: Selective splenic artery angiogram confirms the location of two aneurysms consistent with CT angiogram. C: transarterial splenic artery embolization was carried out with 11 steel coils (5–15 mm) to completely occlude the main splenic artery and two aneurysms.

After 1 week, the patient had a second episode of haematemesis of 200 mL of bright red blood. An emergency TIPS procedure was carried out. First, percutaneous transhepatic occlusion of coronary vein and short gastric vein was performed to treat esophagogastric variceal hemorrhage; then, shunt was created between the middle hepatic vein and left portal vein. One 8 × 60 mm bare stent (E-Luminexx, Bard Peripheral Vascular, Tempe, AZ, USA) and another 8 × 40 mm covered stent (Flucency, Bard Peripheral Vascular, Tempe, AZ, USA) were implanted to complete the shunt. The portosystemic gradient was reduced from 20 to 11 mm Hg. Two week later, transarterial splenic artery embolization was carried out with the objective of complete occlusion of the main splenic artery and two SAAs (Fig. 4). Successful embolization required 11 steel coils (5–15 mm). The patient was commenced on aspirin (100 mg/day) and discharged the next week.

**Figure 5:**
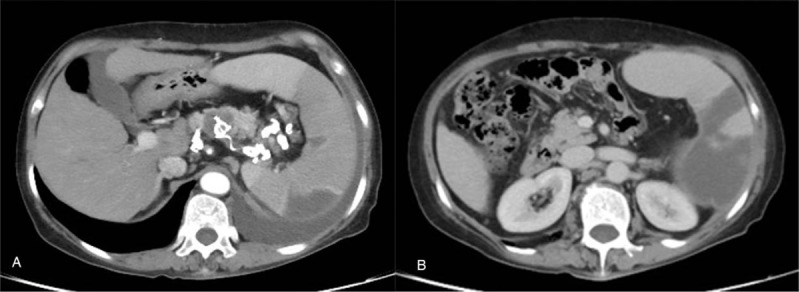
Follow-up enhanced CT was achieved after 3 months following embolization. A: Arterial phase shows complete devascularization of the main splenic artery and two aneurysms, and partial coils protrude into aneurysm sac. B: Venous phase shows segmental splenic infarcts (approximately 50% volume).

She was reviewed 3 months after the operation. Laboratory tests revealed normal leukocyte (5.32 × 10/L), hemoglobin (112 g/L) and platelet counts (167 × 10/L). Liver function grade was classified into Child A. Contrast enhanced CT confirmed patency of the shunt, complete occlusion of the main splenic artery from the splenic hilum to the proximal, and thrombosis of the two aneurysm sac. The majority of the splenic parenchyma was well perfused, with around 50% estimated loss (Fig. 5).

The study was approved by medical ethical research committee in our hospital and the ethical approval number is 2019B003. Informed consent was obtained from the patient for publication of this case report and any accompanying images.

The patient gave consent to publish this case report and read the article and confirmed its content.

## 3 Discussion

SAAs are the commonest visceral artery aneurysms, constituting 50% to 70% of these aneurysms.^[4]^ The pathogenesis of SAA remains unclear, the most probable mechanism include loss of the media layer characterized by disintegration of elastic fibers and smooth muscles.^[5]^ The possible risk factors for SAAs are hypertension, atherosclerosis, cirrhosis, PHT, liver transplantation, female, pregnancy, and multiparity.^[6,7]^ Pregnancy and PHT are the most important factors. About 7% to 50% of patients with SAA have cirrhosis and PHT, while PHT is diagnosed in 50% of SAA patients.^[8]^ Spontaneous SAA rupture is the most life-threatening complications, incidence of spontaneous rupture of a SAA is 2% to 10%, and the mortality rate following rupture is 10% to 40%.^[5,9]^ Pregnancy, clinically symptomatic aneurysm, diameter ≥2 cm, increase in diameter, surgical treatments influencing portal system pressure, PHT, and liver transplantation are among the leading risk factors for rupture.^[10]^

There are rarer reports about multiple SAAs with PHT that was treated with combined TIPS and coil embolization. Only one analogous literature reported a patient, who had undergone a TIPS procedure for PHT complicating cystic fibrosis 18 months previously, was found multiple SAAs located in the splenic parenchyma, and embolized with steel coils and embospheres microspheres.^[11]^ In our report, the patient was admitted to emergency room with hemorrhage of oesophageal varices. At first, we successfully controlled threatening-life bleeding by endoscopic hemostasis and TIPS procedure. Upon admission, emergency US revealed SAA in the proximal of splenic artery, but missed another smaller SAA near the splenic hilum. As an economic and convenient diagnostic tool, US is the first choice, but obesity, gas artefacts and a relatively lower sensitivity for smaller aneurysms are disadvantages of US.^[12]^ CT angiogram accurately provide location and dimension of two SAAs. Treatment is recommended for asymptomatic patients with lesions with dimensions ≥2 cm, who are pregnant or fertile, have PHT, or are candidates for liver transplantation.^[6,12–14]^ The main treatment options for SAAs include open surgery(open or laparoscopic), endovascular treatment (coil embolization or stent), which is becoming more popular, and medical treatment.^[9,13]^ Considering the high rupture risk of aneurysm and poor physical condition, endovascular treatment becomes currently the first option for the patient. Endovascular treatment encompasses implantation of a covered-stent or coil embolization. Coil embolization of main splenic artery is not only more economical procedure than implantation of covered-stent, but also can relieve critical hypersplenism symptoms and easily treat the lesion located in the splenic hilum. Current practice is to deploy 11 different dimensional coils both distal and proximal to the splenic artery to obtain complete occlusion of the main splenic artery. The most frequent complication of transcatheter embolization are splenic infarct. Due to the collateral blood supply to the spleen, complete splenic infarction is relatively rare, but some degree of ischemia is common.^[15]^ Presumably, blood supply through the short gastric arteries is sufficient to preserve the spleen in most cases. In one series of 16 patients treated for SAA with coil embolization there were no complete infarctions but 44% suffered partial splenic infarction. The proportion of the spleen involved varied from 30% to 80%, with a mean of 54%.^[16]^ In our case, 3 month follow-up enhanced CT confirmed complete occlusion of the main splenic artery, thrombosis of the two aneurysm sac, multiple wedge-shaped splenic infarctions with around 50% estimated loss. Laboratory tests revealed complete remission of hypersplenism. Those proved splenic arterial embolization, as a more economical and less invasive procedure, can successfully achieve permanent thrombosis of aneurysm sac, and reserve partial splenic function.

In conclusion, PHT is either a significant risk factor of development of SAAs, or predisposing factor of rupture. In this report here, TIPS can control easophagogastric variceal hemorrhage and decrease portal venous pressure, coil embolization of the main splenic artery can promote permanent thrombosis of aneurysm sac and relieve hypersplenism.

## Author contributions

**Conceptualization:** Niu Hongtao.

**Investigation:** Sha Junfeng, Zou Zibo.

**Resources:** An Jianli.

**Writing – original draft:** Dong Yanchao.
